# Automated Detection and Measurement of Dural Sack Cross-Sectional Area in Lumbar Spine MRI Using Deep Learning

**DOI:** 10.3390/bioengineering10091072

**Published:** 2023-09-10

**Authors:** Babak Saravi, Alisia Zink, Sara Ülkümen, Sebastien Couillard-Despres, Jakob Wollborn, Gernot Lang, Frank Hassel

**Affiliations:** 1 Department of Orthopedics and Trauma Surgery, Medical Center—University of Freiburg, Faculty of Medicine, University of Freiburg, 79106 Freiburg, Germany; sara.uelkuemen@hotmail.de (S.Ü.); gernotmichaellang@gmail.com (G.L.); 2 Department of Spine Surgery, Loretto Hospital, 79100 Freiburg, Germany; alisia.zink@gmail.com (A.Z.); frank.hassel@rkk-klinikum.de (F.H.); 3 Institute of Experimental Neuroregeneration, Spinal Cord Injury and Tissue Regeneration Center Salzburg (SCI-TReCS), Paracelsus Medical University, 5020 Salzburg, Austria; s.couillard-despres@pmu.ac.at; 4 Department of Anesthesiology, Perioperative and Pain Medicine, Brigham and Women’s Hospital, Harvard Medical School, Boston, MA 02115, USA; jwollborn@bwh.harvard.edu; 5 Austrian Cluster for Tissue Regeneration, 1200 Vienna, Austria

**Keywords:** lumbar spine MRI, dural sack cross-sectional area, deep learning, automated detection, spinal pathologies, image segmentation, spine surgery, quantitative measurement, clinical application

## Abstract

Lumbar spine magnetic resonance imaging (MRI) is a critical diagnostic tool for the assessment of various spinal pathologies, including degenerative disc disease, spinal stenosis, and spondylolisthesis. The accurate identification and quantification of the dural sack cross-sectional area are essential for the evaluation of these conditions. Current manual measurement methods are time-consuming and prone to inter-observer variability. Our study developed and validated deep learning models, specifically U-Net, Attention U-Net, and MultiResUNet, for the automated detection and measurement of the dural sack area in lumbar spine MRI, using a dataset of 515 patients with symptomatic back pain and externally validating the results based on 50 patient scans. The U-Net model achieved an accuracy of 0.9990 and 0.9987 on the initial and external validation datasets, respectively. The Attention U-Net model reported an accuracy of 0.9992 and 0.9989, while the MultiResUNet model displayed a remarkable accuracy of 0.9996 and 0.9995, respectively. All models showed promising precision, recall, and F1-score metrics, along with reduced mean absolute errors compared to the ground truth manual method. In conclusion, our study demonstrates the potential of these deep learning models for the automated detection and measurement of the dural sack cross-sectional area in lumbar spine MRI. The proposed models achieve high-performance metrics in both the initial and external validation datasets, indicating their potential utility as valuable clinical tools for the evaluation of lumbar spine pathologies. Future studies with larger sample sizes and multicenter data are warranted to validate the generalizability of the model further and to explore the potential integration of this approach into routine clinical practice.

## 1. Introduction

Lumbar spine magnetic resonance imaging (MRI) plays a pivotal role in the diagnosis and treatment planning of various spinal pathologies, such as degenerative disc diseases, spinal stenosis, and herniated discs [1]. Among these, the assessment of the dural sack cross-sectional area (DSCA) is crucial for evaluating the severity of spinal canal narrowing and associated symptoms [2]. Traditionally, the measurement of DSCA relies on manual segmentation by radiologists, which is a time-consuming and labor-intensive process prone to inter- and intra-observer variability [3].

In recent years, deep learning techniques, particularly convolutional neural networks (CNNs), have shown remarkable potential in medical image analysis, including segmentation, classification, and detection tasks [4]. The U-Net architecture, a widely adopted CNN for biomedical image segmentation, has demonstrated success in various applications, such as cell segmentation, organ segmentation, and tumor detection [5]. However, the automated detection and calculation of DSCA in lumbar spine MRI using deep learning models have not been explored yet. Several studies have addressed the application of deep learning techniques in spinal MRI analysis [6]. For instance, researchers have used CNNs to detect lumbar disc degeneration, vertebral fractures, and spinal metastases [6]. Despite the growing interest in automating spinal MRI analysis, there are a limited number of studies specifically focusing on DSCA measurement. Given the importance of DSCA in the assessment of spinal canal narrowing and its clinical implications, developing an automated method for accurate and efficient DSCA calculation is of high significance.

Current manual methods of DSCA measurement are not only time-consuming but also subject to variability, as different radiologists may produce varying results [7]. An automated deep learning-based approach has the potential to address these limitations, offering consistent and objective measurements that can ultimately improve diagnostic accuracy and treatment planning. Furthermore, such a method could reduce the burden on radiologists, allowing them to focus on more complex and demanding tasks.

In this study, we aimed to develop deep learning algorithms to automate the detection and quantification of DSCA in axial T1-weighted lumbar spine MRI in one setting. We trained and validated our model on a publicly available lumbar spine MRI dataset and further assessed its performance using an external dataset obtained from our hospital. Our aim was to test the feasibility and effectiveness of the deep learning models in automating DSCA measurement, paving the way for its potential integration into clinical practice. By introducing this automated approach to DSCA assessment, we hope to contribute to the growing body of research on deep learning applications in medical imaging. This study also provides valuable insights into the development of deep learning models for the detection and quantification of other clinically relevant features in spinal MRI. Ultimately, we aimed to improve the efficiency and accuracy of spinal pathology assessment, leading to better treatment planning and patient outcomes.

## 2. Materials and Methods

### 2.1. Data Handling

An online repository was used for the development of the algorithm [8,9]. The lumbar MRI dataset consisted of 515 symptomatic back pain patients with diverse demographics. Each patient’s MRI scans included sagittal and axial views of the lowest three vertebrae and discs. The majority of image slices had a 320 × 320 pixel resolution with 12-bit per pixel precision. T1-weighted MRI images were used from this dataset. The ground truth, essential for training and testing image segmentation machine learning algorithms, comprises labeled images marking the dural sack (region of interest (ROI)). The labeling process involved the use of expert-labeled examples to train non-experts, who then labeled the remaining dataset under supervision. The expert radiologist selected the best axial-view slices for the last three segments. The best axial-view slice was identified as the one that intersects closest to the median height of the disc. The external validation dataset (n = 50 patients) was obtained from the Department of Spine Surgery at Loretto-Hospital Freiburg, an affiliated hospital of the University Medical Center Freiburg. The study included consecutive patients who underwent surgical procedures between 2016 and 2021 for lumbar spinal stenosis. The dataset was part of a previous retrospective observational study [10,11] and was approved by the local Ethics Committee of Freiburg, Germany (Number: 116/200). Informed written consent was obtained from each patient prior to their participation in the study. Lumbar T1-weighted MRI dicom data were used for analysis and imported into 3D Slicer software (version: 5.2.2) [12]. A semiautomatic method was employed by manually defining the central parts of the dural sack and obtaining the local intensity histograms in order to segment the dural sack. In the next step, these thresholding values were used for growing volumetric segmentation and its adjacent slices. A subsequent manual adjustment was performed thereafter. The segmentation involved 3–8 slices of the lumbar segments per patient.

### 2.2. Deep Learning Model and Analyses

The dataset was acquired from T1-weighted axial MRI scans and manually segmented labels (ground truth). Images were utilized with 320 × 320 pixel resolution and normalized by dividing each pixel intensity by 255.0. The label images were binarized, setting the pixels with a value of 150/255 to 1.0 and the rest to 0.0. The dataset was subjected to a 5-fold cross-validation, ensuring a robust evaluation of the models’ performance across different subsets of the data, providing a more generalized and accurate measure of their effectiveness. Each of the three deep learning models (U-Net, Attention U-Net, and MultiResUNet) was trained and validated five times, with each iteration serving as the validation set once, while the remaining four iterations formed the training set.

**Figure 1:**
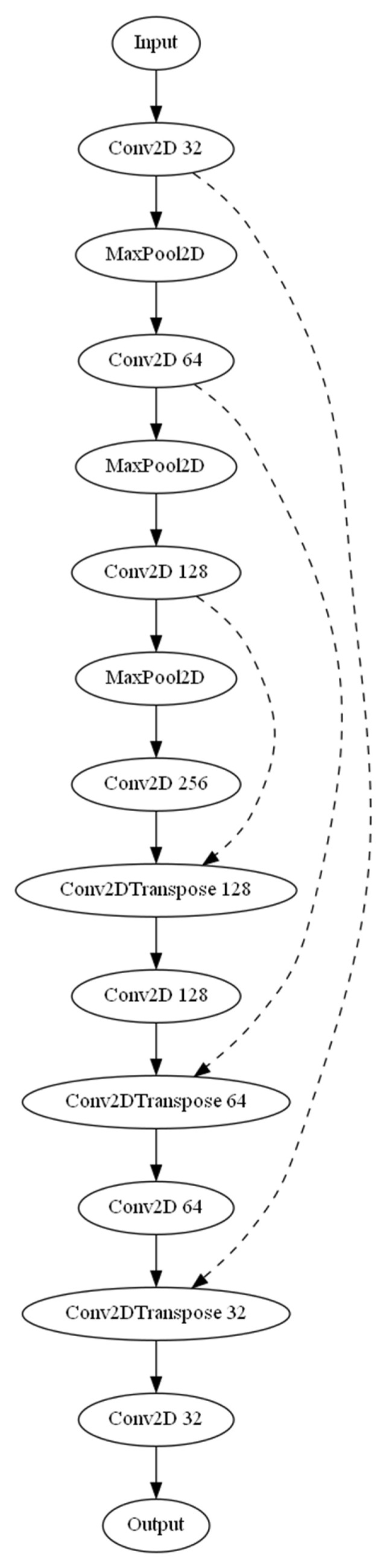
Illustration of the U-Net architecture used for image segmentation. The network is organized in a sequential structure and consists of an encoding path and a decoding path. The encoding path includes a series of convolutional layers (Conv2D) followed by max-pooling layers (MaxPooling2D) to reduce the spatial dimensions and increase the number of feature channels. The decoding path includes a series of transposed convolutional layers (Conv2DTranspose) followed by concatenation operations (indicated by arrows on the right) to combine the output with the corresponding feature maps from the encoding path. Finally, a series of convolutional layers are applied to refine the output. The network output is a single-channel image representing the segmentation mask obtained using a sigmoid activation function in the last convolutional layer.

Three separate architectures were employed for the task of automated detection and calculation of the dural sack cross-sectional area. The base U-Net model (Figure 1), an Attention U-Net variant, and a MultiResUNet were used. The three models were compiled using the Adam optimizer along with a custom-weighted binary cross-entropy loss function. The weights for the positive and negative classes were set as 20.0 and 1.0, respectively. The base U-Net model incorporated several layers, including convolutional, max pooling, and up-sampling layers, with ReLU activation functions. The U-Net model was enhanced by introducing attention gates in the Attention U-Net model to selectively emphasize certain features during training, improving the model’s ability to focus on specific areas of the images. The MultiResUNet model leverages the concept of multi-resolution analysis, which includes a multi-resolution block with three convolution layers and ResPaths for capturing fine details in the image. Each model was trained with a batch size of 32 and over 20 epochs. A ModelCheckpoint callback was used to save the best model based on validation loss. The trained model’s performance was assessed using mean absolute error (MAE) for predicted and ground truth areas. Scatter plots and Bland–Altman plots were generated to visualize the model’s performance. Additionally, a sample image from each model was selected to display the input image, ground truth segmentation, and predicted segmentation, along with the corresponding cross-sectional areas (in mm^2^). The ground truth segmentation cross-sectional area, as annotated by the radiological expert, served as the reference. The calculate_area function was used to compute the cross-sectional area of the dural sack based on the segmentation mask. This function took two inputs: the binary mask of the segmented image and the pixel size in millimeters (obtained from the dicom volume information). This function calculated the number of pixels in the mask using the np.sum function and then multiplied it by the square of the pixel size in millimeters to obtain the area in square millimeters. The output of the function was the calculated area in mm^2^. In order to measure the effectiveness of the employed deep learning models, an array of statistical metrics were computed. The models evaluated included a standard U-Net, an Attention U-Net, and a MultiResUNet model. The model performance was analyzed based on a set of performance metrics, including accuracy, precision, recall, and the F1-score. These metrics provide information about the models’ ability to correctly identify positive instances (precision), their ability to detect actual positives (recall), the overall correctness (accuracy), and the harmonic mean of precision and recall (F1-score). To be specific, accuracy was calculated as the proportion of correct predictions (both positive and negative) to the total number of input samples. Precision was determined as the ratio of true positives to the sum of true and false positives. Recall was calculated as the ratio of true positives to the sum of true positives and false negatives. The F1-score was computed as the harmonic mean of precision and recall, thereby balancing these two values and providing a more comprehensive assessment of model performance.

Beyond these basic metrics, we further analyzed the absolute error in the estimated cross-sectional area of the dural sack in the segmented images. This was computed by comparing the predicted segmentation area to the ground truth. To this end, the mean absolute error (MAE) was computed for each model. MAE provides a measure of the average magnitude of the errors, without considering their direction. MAE provides a linear penalty for each unit of difference between predicted and actual values. The correlation between the actual and predicted dural sack cross-sectional areas was also investigated using the Pearson correlation coefficient. This coefficient measures the linear relationship between two datasets, with a value of +1 indicating a perfect positive correlation, −1 indicating a perfect negative correlation, and 0 implying no correlation. Lastly, Bland–Altman analysis was performed to evaluate the agreement between the ground truth and predicted areas. This involved plotting the difference between the predicted and ground truth areas against the average of these two values, and calculating the mean difference and the limits of agreement (defined as the mean difference ± 1.96 standard deviations). This statistical method is commonly used in medical studies to compare two different measurement techniques.

The resultant statistical evaluation therefore offered a robust understanding of the models’ overall performance in terms of their segmentation accuracy, area estimation, and correlation with ground truth data. This comprehensive analysis allowed for reliable comparisons between the U-Net, Attention U-Net, and MultiResUNet models. The model was further validated on an external dataset, which included both non-segmented and segmented axial MRI scans. The dataset was preprocessed, and the label images were binarized in the same manner as the main dataset. The model’s performance on this external dataset was evaluated as described before. All analyses were conducted in Python. The Python code, including all steps of preprocessing and the algorithm structure, is freely available from the data availability section.

## 3. Results

**Figure 2:**
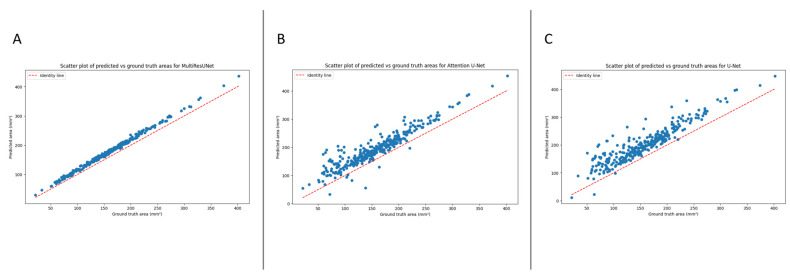
The scatter plot shows the relationship between the ground truth and predicted cross-sectional areas for the dural sack. Each point represents a single image from the validation set (validation set obtained via the best model from k-fold cross validation from sklearn.model_selection in Python). (**A**) MultiResUnet model (Pearson’s correlation coefficient: 0.9980). (**B**) Attention U-Net (Pearson’s correlation coefficient: 0.9264). (**C**) U-Net (Pearson’s correlation coefficient: 0.9196).

The automated segmentation and calculation of the dural sack cross-sectional area (DSCA) displayed a high correlation with the ground truth DSCA across all tested models. The Pearson’s correlation coefficients for U-Net, Attention U-Net, and MultiResUNet were 0.9196, 0.9264, and 0.9980, respectively. This indicates a very strong positive correlation between the predicted and actual areas, suggesting a high degree of accuracy in the segmentation (Figure 2).

Furthermore, these results were externally validated, yielding consistent correlation coefficients for the U-Net, Attention U-Net, and MultiResUNet of 0.9151, 0.9227, and 0.9972, respectively. This reaffirms the robustness and reliability of our models, establishing their potential for accurate DSCA prediction. It is worth noting that the MultiResUNet model outperformed both the U-Net and Attention U-Net models in terms of correlation coefficient, suggesting that it might be more suited to the segmentation and calculation of DSCA in this context. The higher correlation coefficient and lower mean absolute error (MAE) and mean squared error (MSE) of the MultiResUNet model, both in the initial and external validation, indicates superior performance in accurately predicting the DSCA.

**Figure 3:**
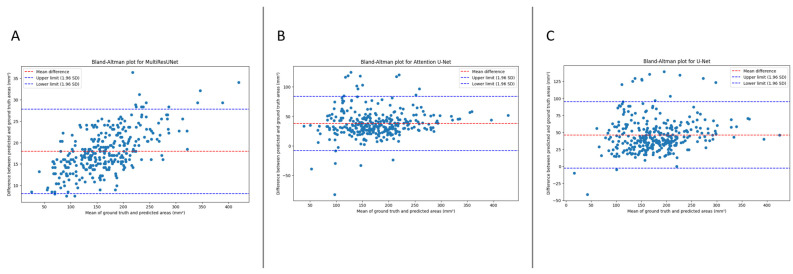
The Bland–Altman plot displays the difference between the predicted and ground truth areas against the mean of the two. The red dashed line signifies the mean difference, with respective values for MultiResUNet, Attention U-Net, and U-Net models indicated. The blue dashed lines represent the limits of agreement, calculated as the mean difference ± 1.96 times the standard deviation of the differences. (**A**) MultiResUNet model (mean difference: 17.9487 mm^2^, limits of agreement: 8.0985 to 27.7989 mm^2^). (**B**) Attention U-Net (mean difference: 38.0006 mm^2^, limits of agreement: −7.8875 to 83.8888 mm^2^). (**C**) U-Net (mean difference: 46.2362 mm^2^, limits of agreement: −2.3701 to 94.8425 mm^2^).

The mean absolute error (MAE) quantifies the model’s performance. This metric denotes the average absolute difference between the predicted and ground truth areas, with lower values indicating superior performance. For our models, the MAEs were as follows: 17.9487 mm^2^ for MultiResUNet, 39.4140 mm^2^ for Attention U-Net, and 46.5972 mm^2^ for U-Net. Figure 3 presents the Bland–Altmann plot, with the limits of agreements being 8.0985 to 27.7989 mm^2^ for MultiResUNet, −7.8875 to 83.8888 mm^2^ for Attention U-Net, and −2.3701 to 94.8425 mm^2^ for U-Net.

The performance of our models was evaluated using various metrics, including accuracy, precision, recall, F1-score, and mean absolute error (MAE). For the main dataset, the MultiResUNet model achieved high accuracy (0.9996) and recall (0.9996) while maintaining an excellent precision (0.8966) and F1-score (0.9453). The MAE was 17.9487 mm^2^, indicating the model’s exceptional ability to estimate the dural sack’s cross-sectional area. The Attention U-Net model achieved an accuracy of 0.9992 and a recall of 0.9903, with a precision of 0.7966 and an F1-score of 0.8829. The MAE was 39.4140 mm^2^. The U-Net model achieved an accuracy of 0.9990 and a recall of 0.9943, with a precision of 0.7673 and an F1-score of 0.8662. The MAE was 46.5972 mm^2^. 

**Figure 4:**
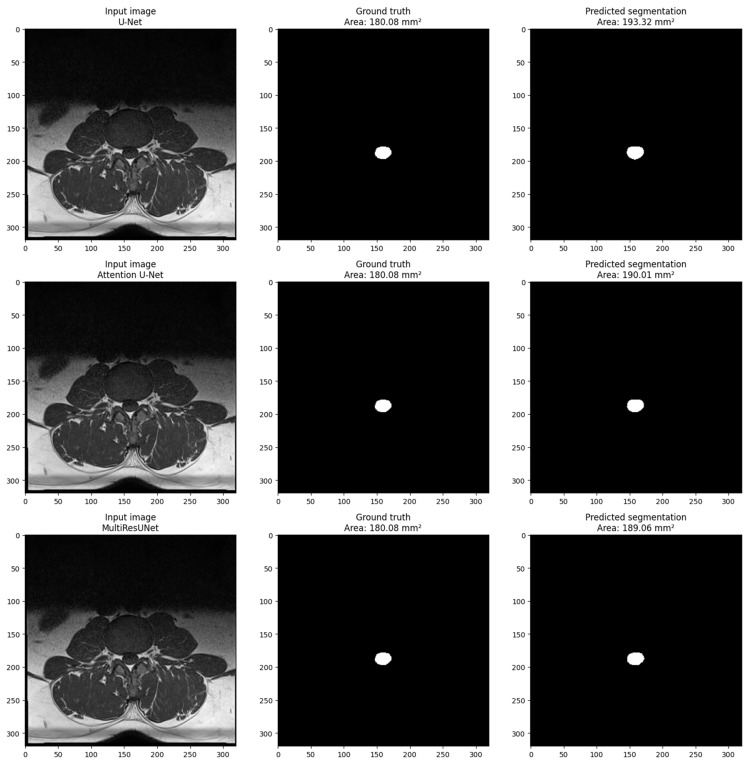
Visualization of a sample from the main dataset (validation set obtained via train_test_split obtained from sklearn.model_selection in Python) for each model (U-Net, Attention U-Net, and MultiResUNet). Each sample includes three images: the input image, the ground truth mask, and the predicted segmentation mask. The pixel size in millimeters is set to 0.6875 based on the volume information provided in the main dataset. The cross-sectional area of the dural sack is calculated for each predicted and ground truth mask using the calculate_area() function. The calculated areas are displayed in the titles of the ground truth and predicted images.

For the external validation dataset, the MultiResUNet model demonstrated excellent accuracy (0.9995) and precision (0.8862) while maintaining a high recall (0.9989) and F1-score (0.9393). The MAE was 20.7329 mm^2^, indicating a slightly higher error rate compared to the main dataset but still performing very well. The Attention U-Net had an accuracy of 0.9989, a precision of 0.7867, a recall of 0.9871, and an F1-score of 0.8756, with an MAE of 43.4768 mm^2^. The U-Net showed an accuracy of 0.9987, a precision of 0.7545, a recall of 0.9912, and an F1-score of 0.8553, with an MAE of 50.8731 mm^2^. Despite the differences in performances, all models demonstrated potential for clinical application when further trained with larger datasets. Figure 4 shows the visualization of a sample from the main dataset (validation set obtained via train_test_split obtained from sklearn.model_selection in Python) for each model. The sample includes three images: the input image, the ground truth mask, and the predicted segmentation mask. The pixel size in millimeters is set to 0.6875 based on the volume information provided in the main dataset volume information. The cross-sectional area of the dural sack is calculated for each predicted and ground truth mask using the calculate_area() function. The calculated areas are displayed in the titles of the ground truth and predicted images. The visualization shows that the model performs well in segmenting the dural sack region in the input images. The predicted segmentation masks closely resemble the ground truth masks, as evidenced by the similarity in the cross-sectional areas calculated for each. 

## 4. Discussion

This study developed a deep learning-based method for detecting and quantifying the dural sack cross-sectional area (DSCA) in axial T1-weighted lumbar spine MRI images. Manually measuring DSCA is a time-consuming process that can be subject to errors due to differences between and within observers. The developed models, including the U-Net, Attention U-Net, and MultiResUNet, all showcased remarkable performance. The MultiResUNet model outperformed the others, demonstrating exceptional accuracy and recall on the main dataset and outstanding precision on the external validation dataset. The U-Net and Attention U-Net models also demonstrated commendable performance, with high accuracy and recall values on both datasets. Considering the limited amount of data used for training, further training of the models can provide consistent and objective measurements of DSCA, which can improve diagnostic accuracy and treatment planning, as well as reduce the burden on radiologists.

These results demonstrate that the automatic segmentation and calculation of DSCA using deep learning models is a feasible and effective method. Each of the models—the U-Net, Attention U-Net, and MultiResUNet model—delivered robust results with strong correlations between the ground truth and predicted DSCA, indicating their precision. The mean absolute error (MAE), signifying the average absolute disparity between the predicted and ground truth areas, varied across the models. For the MultiResUNet model, the most accurate model, the MAE was 17.9487 mm^2^ for the main dataset and 20.7329 mm^2^ for the external validation dataset. Despite the slight increment in MAE for the external validation dataset, the model’s performance underscores its potential for broad application and clinical utility. The U-Net and Attention U-Net models, while slightly less precise, still showcased satisfactory performance, with respective MAEs for the main dataset of 46.5972 mm^2^ and 39.4140 mm^2^, and for the external validation dataset of 50.8731 mm^2^ and 43.4768 mm^2^. These results emphasize the reliability of these models and their prospective value in clinical settings.

There have been several previous studies on automatic segmentation and detection in a variety of medical fields, including spine research. Yet, only a few studies have provided an approach to automatic segmentation and calculation of the dimensions of segmented structures. To our knowledge, there has been no study to date that has presented an algorithm for the automated artificial intelligence-based segmentation and calculation of spine structures in the same context. Using two fully convolutional neural networks (CNNs) based on SegNet and U-Net, Adoui et al. (2019) proposed two deep learning approaches for automating breast tumor segmentation in dynamic contrast-enhanced magnetic resonance imaging (DCE-MRI) [13]. In another study, Wu et al. (2023) described a two-step segmentation-based deep learning approach to detect adolescent scoliosis using augmented U-Nets with non-square kernels [14]. Studies such as these illustrate the potential of deep learning models, including U-Net architectures, in the segmentation and detection of medical images.

It is noteworthy that only one similar study has been published previously. El Mendili et al. developed a semiautomatic algorithm for calculating the cross-sectional area of the dural sack in the cervical spine [7]. Unlike our study, which focused on the cross-sectional area of the dural sack in lumbar spine MRI scans, this study aimed to segment the spinal cord in cervical and thoracic MRI scans. Additionally, whereas our study employed deep learning-based methods, El Mendili et al. employed a semi-automated double-threshold-based approach. Deep learning-based methods are particularly advantageous due to their ability to learn from large datasets and generalize to new data, whereas semi-automated methods such as those presented by El Mendili et al. may not scale to larger datasets due to the requirement for manual intervention. In terms of the results, both studies achieved high accuracy in their respective regions of interest. For the cross-sectional area of the dural sack in the lumbar region, our study achieved varying results across different models. The best-performing model, MultiResUNet, achieved an absolute error of 17.9487 mm^2^ and an accuracy of 99.96% for the main dataset. The U-Net and Attention U-Net models, while slightly less precise, had absolute errors of 46.5972 mm^2^ and 39.4140 mm^2^ and accuracies of 99.90% and 99.92%, respectively. El Mendili et al., using the proposed semi-automated double threshold-based method and the Dice similarity coefficient as evaluation metrics for segmentation, achieved a mean Dice similarity coefficient of 95.71% for cervical segmentation and 94.27% for thoracic segmentation. In summary, both studies have demonstrated the potential of (semi)automated segmentation methods to improve diagnostic accuracy and reduce the burden placed upon radiologists. Further research is needed to compare the performance of both methods on larger and more diverse datasets, as well as to examine their potential clinical implications.

Some limitations are associated with this study. First, the model was trained and validated on a limited set of data. The generalizability of our method needs to be confirmed using larger datasets from different institutions. Second, the calculation of DSCA using this method is limited to T1-weighted axial lumbar spine MRIs. While these images offer superior anatomical detail and are less susceptible to artefacts compared to T2-weighted images, it is worth noting that T2-weighted images generally provide clearer delineation of the thecal sac due to their high signal intensity in cerebrospinal fluid. The use of T1-weighted images was driven primarily by the availability of the labeled dataset, and future work integrating T2-weighted images could potentially enhance the accuracy of our model. In order to extend our method to other imaging modalities and anatomical regions, further research is required. An additional limitation of our study is the unavailability of demographic data for the main dataset obtained from the Lumbar Spine Research database. This precluded us from making a comprehensive comparison with the external validation dataset, which comprised 31 males and 19 females with a mean age of 56.46 years. Such comparisons could have provided insights into the influence of demographic variables on the model’s performance, and we acknowledge the need for further studies using datasets with complete demographic information. Furthermore, we only focused on DSCA detection and quantification. A further investigation is required to assess the potential clinical implications of automated DSCA measurement using deep learning models for other anatomical structures. 

Although our study has several limitations, it contributes to the growing body of research on deep-learning applications in medical imaging [6]. DSCA is a crucial parameter for evaluating spinal canal narrowing and associated symptoms in lumbar spine MRI, and our approach is unique in that it focuses specifically on the automated detection and quantification of DSCA in lumbar spine MRI. U-Net-based deep learning models achieved high accuracy and recall on the main dataset, which indicates its potential for clinical application. Aside from its potential clinical impact, the presented method also offers practical advantages. By automating the detection and quantification of DSCA, radiologists will be able to reduce their workload and improve diagnostic efficiency. Taking into account the aging population and the rising demand for diagnostic imaging studies, this is of particular importance in this regard. Additionally, this study contributes to a broader discussion on the use of deep learning models in medical image analysis. As noted, deep learning techniques have shown remarkable potential in medical image analysis, and U-Net architectures, in particular, have been successfully applied in various applications such as cell segmentation, organ segmentation, and tumor detection [5]. The results of our study demonstrate that the presented architectures based on U-Net can also be applied to the automated detection and quantification of DSCA in lumbar spine MRIs.

## 5. Conclusions

In conclusion, this study demonstrates the potential of deep learning models, specifically U-Net-based architectures, for the automated detection and calculation of DSCA in axial T1-weighted lumbar spine MRI. The proposed models achieved high accuracy and recall, and our results indicate their potential for clinical application. The automated approach to DSCA measurement has the potential to offer consistent and objective measurements, improve diagnostic accuracy and treatment planning, and reduce the burden on radiologists. Future studies are needed to validate our method on larger datasets and explore its clinical implications.

## Data Availability

The Python codes and algorithm structures are available from: https://github.com/Freiburg-AI-Research (accessed on 6 September 2023).
